# Long-Term Liver Expression of an Apolipoprotein A-I Mimetic Peptide Attenuates Interferon-Alpha-Induced Inflammation and Promotes Antiviral Activity

**DOI:** 10.3389/fimmu.2020.620283

**Published:** 2021-02-23

**Authors:** Myriam Fernandez-Sendin, Claudia Augusta Di Trani, Angela Bella, Marcos Vasquez, Nuria Ardaiz, Celia Gomar, Leire Arrizabalaga, Sergio Ciordia, Fernando J. Corrales, Fernando Aranda, Pedro Berraondo

**Affiliations:** ^1^ Program of Immunology and Immunotherapy, Cima Universidad de Navarra, Pamplona, Spain; ^2^ Navarra Institute for Health Research (IDISNA), Pamplona, Spain; ^3^ Functional Proteomics Laboratory, National Center for Biotechnology, Consejo Superior de Investigaciones Científicas (CSIC), Madrid, Spain; ^4^ Centro de Investigación Biomédica en Red de Enfermedades Hepáticas y Digestivas (CIBEREHD), Madrid, Spain; ^5^ Centro de Investigación Biomédica en Red de Cáncer (CIBERONC), Madrid, Spain

**Keywords:** liver, antiviral, apolipoprotein A-I mimetic peptide, type I Interferon, adeno-associated virus

## Abstract

Apolipoprotein A-I mimetic peptides are amphipathic alpha-helix peptides that display similar functions to apolipoprotein A-I. Preclinical and clinical studies have demonstrated the safety and efficacy of apolipoprotein A-I mimetic peptides in multiple indications associated with inflammatory processes. In this study, we evaluated the effect of the long-term expression of L37pA in the liver by an adeno-associated virus (AAV-L37pA) on the expression of an adeno-associated virus encoding interferon-alpha (AAV-IFNα). Long-term IFNα expression in the liver leads to lethal hematological toxicity one month after AAV administration. Concomitant administration of AAV-L37pA prevented the lethal toxicity since the IFNα expression was reduced one month after AAV administration. To identify the mechanism of action of L37pA, a genomic and proteomic analysis was performed 15 days after AAV administration when a similar level of IFNα and interferon-stimulated genes were observed in mice treated with AAV-IFNα alone and in mice treated with AAV-IFNα and AAV-L37pA. The coexpression of the apolipoprotein A-I mimetic peptide L37pA with IFNα modulated the gene expression program of IFNα, inducing a significant reduction in inflammatory pathways affecting pathogen-associated molecular patterns receptor, dendritic cells, NK cells and Th1 immune response. The proteomic analysis confirmed the impact of the L37pA activity on several inflammatory pathways and indicated an activation of LXR/RXR and PPPARα/γ nuclear receptors. Thus, long-term expression of L37pA induces an anti-inflammatory effect in the liver that allows silencing of IFNα expression mediated by an adeno-associated virus.

## Introduction

Apolipoprotein A-I (apoA-I) is the main protein of the high-density lipoproteins. The structure of apoA-I favors binding to phospholipids and cholesteryl esters. ApoA-I is composed of an N-terminal globular domain followed by a tandem array of amphipathic helices (1). ApoA-I formulations have remarkable preclinical activity in models of atherosclerosis and other inflammatory processes, and its efficacy has been evaluated in clinical trials (2). To improve the therapeutic profile of apoAI, apoA-I mimetic peptides have been developed based on this protein’s amphipathic nature. ApoA-I mimetic peptides are synthetic amphipathic peptides that preserve the anti-atherogenic and anti-inflammatory properties of apoA-I. These mimetic peptides bind to oxidized phospholipids with much higher affinity than to non-oxidized lipids (3). Stabilized peptides using D-aminoacids have led to potent drugs evaluated in preclinical models of atherosclerosis (4), diabetes (5), scleroderma (6), arthritis (7), and metabolic syndrome (8), Alzheimer’s disease (9), pulmonary hypertension (10), myocardial infarction (11), inflammatory bowel disease (12) and asthma (13). This intense preclinical research has led to the design of several clinical trials to evaluate the safety and efficacy of the clinical use of apoA-I mimetic peptides. Among the different apoA-I mimetic peptides, we have focused our attention on L37pA. This is an apoA-I mimetic peptide of 18 amino acids with a central proline that divides the peptide structure into two amphipathic helices (14). This peptide can promote the efflux of cholesterol and phospholipids from cells *in vitro*. In vivo, L37pA peptides have been shown to attenuate inflammation in models of cardiac ischemia/reperfusion injury (1) and sepsis (15).

The *in vivo* evaluation of these peptides requires frequent intravenous administrations to achieve therapeutic plasma levels. To improve stability, D-aminoacids have been introduced into the chemical structure of the peptides rendering them resistant to the proteases present in plasma. As a result of the increased stability, the half-life in circulation is extended. An alternative approach to overcome the pharmacokinetic limitations of peptides is the use of gene therapy vectors. Among these, adeno-associated (AAV) vectors are being widely used in preclinical research and clinical trials due to their unique properties as gene therapy vectors. AAV are non-pathogenic viruses that can be engineered for long-term expression of the transgene of interest. Most of the viral genome is deleted and substituted by an expression cassette of the same size as the deleted viral genome. The expression cassette must contain at least a promoter, the coding sequence of the gene of interest, and a poly-A sequence. This expression cassette must be flanked by the viral inverse terminal repeats. This recombinant viral genome can be packaged into the AAV capsids by cotransfection in HEK293T cells of a plasmid containing the expression cassette and a packaging plasmid that encodes all AAV proteins and proteins of a helper virus required for the recombinant AAV production (16). The clinical relevance of the AAV vectors is illustrated by the FDA-approval of two AAV-based gene therapies: vortigern neparvovec (AAV2-hRPE65v2) for the treatment of patients with RPE65-mediated inherited retinal dystrophy (17, 18) and onasemnogene abeparvovec for spinal muscular atrophy (19). The clinical data that led to the approval of these advanced medical products support the feasibility and safety of gene therapy in humans. However, the fact that 150 clinical trials using adeno-associated are listed in ClinicalTrials.gov is an indication of the numerous hurdles that the AAV drug candidate must face before gaining FDA approval. One of the hurdles that merits more detailed attention is the persistence of AAV expression. Preclinical data in mouse models point to a long-lasting expression for all the animal’s life (20). Reports from clinical trials in humans have shown therapeutic levels of the transgene for up to 10 years in some patients (21), but other clinical trials have revealed silencing in humans (22). This silencing of AAV expression has also been reported in large animal models such as in woodchuck infected with the woodchuck hepatitis virus (23). The immune response against viral capsids has been proposed as the primary mechanism of silencing operating in humans (22, 24). However, AAV silencing remains poorly understood due to the lack of animal models that reproduce this phenomenon. We have previously shown that the simultaneous expression of interferon-alpha (IFNα) and the apoA-I mimetic peptide lead to partial elimination of AAV expression (25). Here, we confirm this finding and performed a genomic and proteomic analysis of the mechanism that mediates AAV silencing.

## Material and Methods

### Production of Recombinant Adeno-Associated Virus Vectors

Experiments were performed with AAV serotype 8 expressing mouse IFNα1 or L37pA under the transcriptional control of the elongation factor 1α promoter (EF). AAV vectors were produced by co-transfection of pDP8.ape (PlasmidFactory GmbH & Co. KG, Bielefeld Germany) and pAAV encoding IFNα or L37pA into HEK-293T cells. For each production, a mix of 20 µg of pAAV, 55 µg of pDP8.ape, and linear PEI MAX 25 kDa (Polysciences, Warrington, PA) was transfected into HEK-293T cells. Two days later, AAV were isolated from the cell lysates by ultracentrifugation in Optiprep Density Gradient Medium (Sigma-Aldrich, St Louis MO). To titer the AAV productions, viral DNA was isolated using The High Pure Viral Nucleic Acid kit (Roche Applied Science. Mannheim, Germany). The concentration of viral particles was subsequently determined by real-time quantitative PCR using primers specific to the EF promoter: Fw: 5’-GGTGAGTCACCCACACAAAGG-3’ and Rv: 5’-CGTGGAGTCACATGAAGCGA-3´.

### Animal Handling

Experiments were performed with 6–8-week-old female C57BL/6 mice with bodyweights between 18–20 g purchased from Harlan Laboratories (Barcelona, Spain). Mice were maintained under pathogen-free conditions and were bred in a temperature-controlled animal facility with a 12 h light-dark cycle. The experimental design was approved by the Ethics Committee for Animal Testing of the University of Navarra. Recombinant AAV8 vectors were administered once *via* intravenous injection in a total volume of 200 µl. Previously, mice were anesthetized with an intraperitoneal injection of ketamine/xylazine mixture, which provides a 10 mg per kg of body weight dose of xylazine and a 100 mg per kg dose of ketamine (dilution 1:10).

### Determination of Interferon-Alpha by ELISA Analysis

IFNα levels were measured using a VeriKine™ Mouse Interferon Alpha ELISA Kit (PBL Assay Science, Piscataway, NJ) following the manufacturer’s recommendations.

### Hemogram

Blood samples were collected on days 30, 60, and 120 after AAV injection in tubes with 0.5% heparin (Mayne Pharma, Mulgrave, Australia) as the final concentration. Hemograms were analyzed using the Drew Scientific HemaVet Hematology Analyzer (CDC Technologies, Oxford, CT) following the manufacturer’s recommendations.

### RNA Isolation and Quantitative RT-PCR Analysis

Total RNA extraction from livers was performed using the Maxwell^®^ 16 Total RNA Purification Kit (Promega, Madison, Wisconsin, USA). The concentration and purity of samples were determined in a NanoDrop spectrophotometer with absorbance set at 260 and 280 nm (Thermo Scientific, Wilmington, USA). Three hundred ng of RNA were retrotranscribed to cDNA with Moloney murine leukemia virus (M-MLV) reverse transcriptase from Promega, according to the manufacturer’s instructions.

Real-time PCR was performed with iQ SYBR Green Supermix (Bio-Rad, Hercules, CA) using specific primers for each gene. Interferon-stimulated gene 15 (ISG15) Fw: 5´- GATTGCCCAGAAGATTGGTG-3´ and Rv: 5´TCTGCGTCAGAAAGACCTCA-3´. Ubiquitin specific peptidase 18 (USP18) Fw: 5´- CCAAACCTTGACCATTCACC-3´ and Rv: 5´- ATGACCAAAGTCAGCCCATCC-3´, 2.5 oligo-adenylate synthetase (2.5 OAS) Fw: 5´- ACTGTCTGAAGCAGATTGCG-3´ and Rv: 5´- TGGAACTGTTGGAAGCAGTC-3´. Serine protease inhibitor A3G (serpina3g) Fw: 5´- CTTCCCAACGGCTGGAATCTA-3´and Rv: 5´- ACTGTCCAATCAGGCATAGCG-. 3´. C-C chemokine receptor type 2 (CCR2) Fw: 5´- atccacggcatactatcaacatc-3´ and Rv: 5´- CAAGGCTCACCATCATCGTAG-3´. C-X-C motif chemokine 10 (CXCL10) Fw: 5´- CCAAGTGCTGCCGTCATTTTC-3´ and Rv: 5´- GGCTCGCAGGGATGATTTCAA-3´. C-X-C motif chemokine ligand 9 (CXCL9) Fw: 5´- GGAGTTCGAGGAACCCTAGTG-3´ and Rv: 5´- GGGATTTGTAGTGGATCGTGC-3´. Ribosomal Protein, Large, P0 (RPLP0), Fw: 5’-AACATCTCCCCCTTCTCCTT-3’ and Rv: 5’-GAAGGCCTTGACCTTTTCAG-3’. As RPLP0 levels remained constant across different experimental conditions, this parameter was used to standardize gene expression. The amount of each transcript was expressed by the formula 2ΔCt [ct(RPLP0) – ct(gene)], ct being the point at which the fluorescence rises significantly above the background fluorescence.

### DNA Isolation and Quantitative PCR Analysis

Total DNA extraction from livers was performed using the QIAamp DNA Mini Kit (Qiagen, Hilden, Germany). The concentration and purity of samples were determined in a NanoDrop spectrophotometer with absorbance set at 260 and 280 nm (Thermo Scientific, Wilmington, USA). Real-time PCR was performed with iQ SYBR Green Supermix (Bio-Rad, Hercules, CA) using specific primers for each gene. EF promotor Fw: 5´- GGTGAGTCACCCACACAAAGG-3´ and Rv: 5´- CGTGGAGTCACATGAAGCGA-3´. Ribosomal Protein Large, P0 (RPLP0), Fw: 5’-AACATCTCCCCCTTCTCCTT-3’ and Rv: 5’-GAAGGCCTTGACCTTTTCAG-3’. As RPLP0 levels remained constant across different experimental conditions, this parameter was used to standardize gene expression. The amount of each transcript was expressed by the formula 2ΔCt [ct(RPLP0) – ct(gene)], ct being the point at which the fluorescence rises significantly above the background fluorescence.

### Histological Analysis

One hundred twenty days after AAV inoculation, mice were euthanized, and their livers were isolated and fixed in 4% of paraformaldehyde for 24 h. Fixed samples were washed with PBS and stored in 70% ethanol at room temperature until processing. Cell infiltration was analyzed histochemically by H&E. Quantification was performed by selecting positive cells for CD3 and F4/80. Pixel intensity and density were quantified over a threshold color using MATLAB software. Optimal cutting temperature compound (Sakura, Torrance, CA)-embedded frozen livers were sectioned at 8 μm and stained with Fat Red (Sigma, St. Louis, MO) according to a standard protocol.

### Biochemical Analysis

One hundred twenty days after AAV inoculation, blood samples were collected, and plasma levels of aspartate aminotransferase (AST) were measured using an automatic biochemical analyzer (Cobas C711, Pamplona, Spain).

### Gene Microarray

RNA was isolated from livers using the Maxwell^®^ 16 Total RNA Purification Kit (Promega) and quantified in a NanoDrop spectrophotometer (Thermo Scientific). Microarray analysis was performed using the Affymetrix platform at Bioinformatics Unit in the CIC-IBMCC. Labeling and hybridizations were performed according to protocols from Affymetrix (Santa Clara, CA). Briefly, 100 ng of total RNA were amplified and labeled using the WT Plus reagent kit (Affymetrix) and then hybridized to Clariom S mouse Array (Affymetrix). Washing and scanning were performed using the GeneChip System of Affymetrix (GeneChip Hybridization Oven 645, GeneChip Fluidics Station 450, and GeneChip Scanner 7G). Both background correction and normalization were performed using the Robust Multi-array Average (RMA) algorithm. After quality assessment, a filtering process was performed to eliminate low expression probe sets. Applying the criterion of an expression value greater than five in at least two samples of one of the experimental conditions, 22,143 probe sets were selected for statistical analysis. Linear Models for Microarray Data (LIMMA) was used to identify the probe sets that showed significant differential expression between each treatment and the untreated mice. Genes were selected as significant using a B-statistic cut-off (B > 0). Functional enrichment analysis of Gene Ontology categories was carried out using a standard hypergeometric test. R and Bioconductor were used for preprocessing and statistical analysis. The functional enrichment analysis was performed using Ingenuity Pathway Analysis (Ingenuity Systems, www.ingenuity.com), whose database includes manually curated and fully traceable data derived from literature sources. Microarray data are available on the Gene Expression Omnibus (GEO) website (accession number: GSE162764).

### Proteomic Analysis

Total protein concentration was determined using the Pierce 660 nm protein assay (Thermo). For digestion, 40 µg of protein from each condition were precipitated by the methanol/chloroform method. Protein pellets were resuspended and denatured in 20 µl 7 M Urea/2M Thiourea/100 mM TEAB, pH 7.5, reduced with 2 µl of 50 mM Tris(2-carboxyethyl) phosphine (TCEP, SCIEX), pH 8.0, at 37°C for 60 min and followed by 1 µl of 200 mM cysteine-blocking reagent (methyl methanethiosulfonate (MMTS, Pierce) for 10 min at room temperature. Samples were diluted up to 140 µl to reduce urea concentrations with 25 mM TEAB. Digestions were initiated by adding 2 µg sequence grade-modified trypsin (Sigma-Aldrich) to each sample in a ratio of 1:20 (w/w), which were then incubated at 37°C overnight on a shaker. Sample digestions were evaporated to dryness in a vacuum concentrator. The resulting peptides were subsequently labelled using the TMT-sixplex Isobaric Mass Tagging Kit (Thermo Scientific, Rockford, IL, USA) according to the manufacturer’s instructions as follows: TMT-1 (126: CTR-1; 127: IFN-1; 128: L37-1; 129: CTR-2; 130: IFN-2; 131: L37-2) and TMT-2 (126: CTR-3; 127: IFN-3; 128: L37-3). After labeling, samples were pooled, evaporated to dryness, and stored at -20°C until the LC−MS analysis. Three biological replicates of each condition were analyzed.

RP C18 fractionation of the TMT labeled peptides was performed using the Pierce™ High pH Reversed-Phase Peptide Fractionation Kit (Thermo Scientific, Rockford, IL, USA). A step gradient of increasing acetonitrile concentrations (5–50% ACN) in a volatile high pH elution solution (0.1% Triethylamine) was applied to the column to elute bound peptides into eight different fractions collected by centrifugation. Finally, fractions were pooled into four fractions using the fraction mixing strategy n+4 (i.e., fractions 1 + 5, 2 + 6, 3 + 7, and 4 + 8). The peptide fractions were dried, desalted using a Stage-Tips C18 (3M), and stored at −20°C until the LC−MS analysis. A 1 µg aliquot of each labeled fraction was subjected to 1D-nano LC ESI-MSMS analysis using a nano liquid chromatography system (Eksigent Technologies nanoLC Ultra 1D plus, SCIEX, Foster City, CA) coupled to high-speed Triple TOF 5600 mass spectrometer (SCIEX, Foster City, CA) with a Nanospray III source. The analytical column used was a silica-based reversed phase Acquity UPLC® M-Class Peptide BEH C18 Column, 75 µm×150 mm, with a 1.7 µm particle size, and 130 Å pore size (Waters). The trap column was a C18 Acclaim PepMapTM 100 (Thermo Scientific), 100 µm×2 cm, 5 µm particle diameter, 100 Å pore size, switched on-line with the analytical column. The loading pump delivered a solution of 0.1% formic acid in water at 2 µl/min. The nano-pump provided a flow-rate of 250 nl/min and was operated under gradient elution conditions. Peptides were separated using a 150 min gradient ranging from 2 to 90% mobile phase B (mobile phase A: 2% acetonitrile, 0.1% formic acid; mobile phase B: 100% acetonitrile, 0.1% formic acid). Injection volume was 5 µl.

Data acquisition was performed with a TripleTOF 5600 System (SCIEX, Foster City, CA). Data were acquired using an ionspray voltage floating (ISVF) 2300 V, curtain gas (CUR) 35, interface heater temperature (IHT) 150, ion source gas 1 (GS1) 25, declustering potential (DP) 150 V. All data were acquired using information-dependent acquisition (IDA) mode with Analyst TF 1.7 software (SCIEX, Foster City, CA). For IDA parameters, 0.25 s MS survey scan in the mass range of 350–1,250 Da were followed by 30 MS/MS scans of 150 ms in the mass range of 100–1,800. Switching criteria were set to ions greater than mass to charge ratio (m/z) 350 and smaller than m/z 1250 with charge state of 2–5 and an abundance threshold of more than 90 counts (cps). Former target ions were excluded for 20 s. IDA rolling collision energy (CE) parameters script was used for automatically controlling the CE.

The mass spectrometry data obtained were processed using PeakView^®^ 1.2 Software (SCIEX, Foster City, CA) and exported as mgf files. Proteomics data analyses were performed by using four search engines (Mascot, OMSSA, X!Tandem and Myrimatch) and a target/decoy database built from sequences in the Mus musculus proteome in the Uniprot Knowledgebase. Search engines were configured to match potential peptide candidates with a mass error tolerance of 25 ppm and a fragment ion tolerance of 0.02Da, allowing for up to two missed tryptic cleavage sites and a maximum isotope error (13C) of one, considering fixed MMTS modification of cysteine and variable oxidation of methionine, pyroglutamic acid from glutamine or glutamic acid at the peptide N-terminus, and modification of lysine and peptide N-terminus with TMT 6-plex reagents. Score distribution models were used to compute peptide-spectrum match p-values (26), and spectra recovered by a FDR* <=0.01 (peptide-level) filter were selected for quantitative analysis. Approximately 5% of the signals with the lowest quality were removed prior to further analysis. Differential regulation was measured using linear models (27), and statistical significance was measured using q-values (FDR). All analyses were conducted using software from Proteobotics (Madrid, Spain). Proteomics data have been deposited to the ProteomeXchange Consortium via the PRIDE partner repository with the dataset identifier PXD023281.

### Plasma Lipoprotein Profile

Plasma lipoproteins were fractionated using fast protein liquid chromatography. A Superose (Pharmacia, Herts, UK) column was used. A 200 µl sample of plasma pooled from three mice was injected, and then eluted with phosphate-buffered saline at a flow rate of 0.4 ml per min. Fractions (0.25 mL) were collected. Cholesterol was determined using an automatic biochemical analyzer (Cobas C711).

### Statistical Analysis

GraphPad Prism version 8.2.1 software (GraphPad Software, Inc., San Diego, CA) was used for statistical analysis. P values <0.05 were considered to be statistically significant. The analysis used in each case is indicated in the figure legends.

## Results

### Coadministration of Adeno-Associated Virus-L37pA Prevents the Lethal Toxicity of Adeno-Associated Virus-Interferon-Alpha

**Figure 1:**
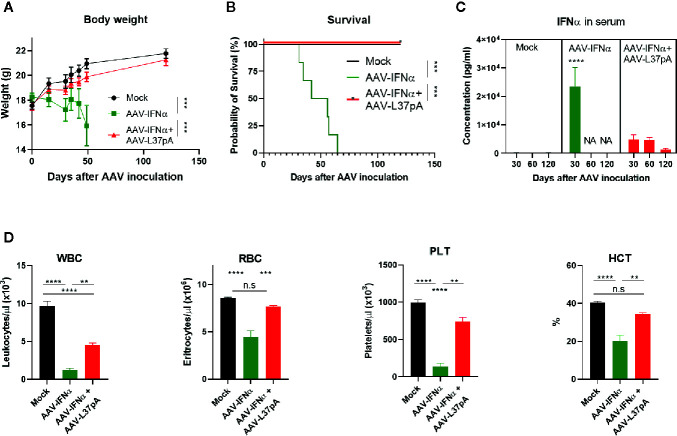
Coadministration of AAV-L37 avoids the lethal toxicity induced by AAV-IFNα. C57BL/6 mice (N = 6) were left untreated (Mock) or received 1.2x10^11^ vg/mouse of AAV-IFNα alone (AAV-IFNα) or combined with 9.5x10^11^ vg/mouse AAV-L37pA and the body weight **(A)** and survival **(B)** was followed up for 120 days. At day 30, 60, and 120, serum levels of IFNα were determined by ELISA **(C)** and complete blood counts were analyzed with a Hemavet Hematology Analyzer. **(D)** WBC, white blood cells; RBC, red blood cells; PLT, platelets; HCT, hematocrit. Mean ± SEM is represented. **p < 0.01 ***p < 0.001 ****p < 0.0001. Body weight was analyzed by Extra Sum-of-Square F test after non-linear regression with a third order polynomial least square fit. Survival was analyzed by Log-Rank test. IFNα concentrations were analyzed by nested one-way ANOVA followed by Dunnett’s multiple comparisons test. **** indicate p < 0.0001 for AAV-IFNα compared to the other two experimental groups. Red blood counts were analyzed by one-way ANOVA followed by Tukey’s multiple comparisons test.

AAV vectors allow stable long-term expression of the transgene in the liver. In the case of IFNα, sustained expression of low levels of this cytokine reduced the bodyweight of treated mice gradually (**Figure 1A**). Mice from the AAV-IFNα group started to die after one month after injection and all mice from the AAV-IFNα groups had to be sacrificed after 65 days (**Figure 1B**). On day 30, the serum IFNα levels in this experimental group were higher than 20 ng/ml (**Figure 1C**) and induced hematological toxicity characterized by a drop in circulating levels of white blood cells, red blood cells, platelets, and low hematocrit (**Figure 1D**). Coadministration of an AAV vector encoding the apolipoprotein A-I mimetic peptide L37pA (AAV-L37pA) normalized most of the analyzed parameters. Bodyweight and survival followed the same trend as non-treated mice (Mock) (**Figures 1A, B**). Serum levels of IFNα were detectable at day 30, 60, and 120 but were fivefold lower than IFNα levels in AAV-IFNα treated mice at day 30. IFNα levels in the group treated with the AAV combination remained constant between days 30 and 60, but at day 120, serum levels decreased fourfold (**Figure 1C**). The lack of lethal toxicity and reduced circulating IFNα was reflected in normal red blood levels and hematocrit, and lower reductions of white blood cells and platelets than those observed with the AAV-IFNα alone (**Figure 1D**).

**Figure 2:**
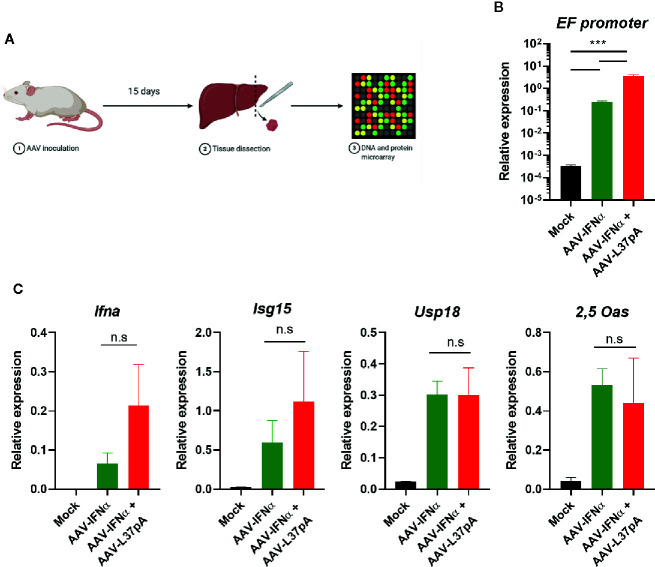
Comparable AAV infection and expression IFN related genes in the liver two weeks after AAV administration. C57BL/6 mice (N = 3) were left untreated (Mock) or received 1.2x10^11^ vg/mouse of AAV-IFNα alone (AAV-IFNα) or combined with 9.5x10^11^ vg/mouse AAV-L37pA. Two weeks later, mice were sacrificed and DNA, RNA and proteins from livers were purified. **(A)** Schematic representation of the experimental setting. **(B)** AAV viral genome was quantified by quantitative PCR of total DNA. **(C)** The expression of the AAV transgene mIFNα and the interferon-stimulated genes, interferon-stimulated gene 15 (ISG15), ubiquitin-specific peptidase 18 (USP18), and 2’-5’-oligoadenylate synthetase (2,5-OAS) were determined by RT-qPCR. Mean ± SEM is represented. n.s, non-significant; ***p < 0.001 One-way ANOVA followed by Tukey’s multiple comparisons test.

To elucidate the mechanism that mediated the transgene expression’s abrogation produced by the combination of AAV-IFNα and AAV-L37pA, mice treated with AAV-IFNα, with the AAV combination or non-injected mice were sacrificed at an early timepoint (**Figure 2A**) when the AAV genome was similar in both experimental groups. The amount of AAV genome in the liver was determined by quantitative real-time PCR using primers designed to amplify a fragment of the EF1α promoter included in both AAVs. At this early time point, the PCR signal was three orders of magnitude higher than the non-specific signal of non-injected mice. Mice treated with the AAV combination received a double amount of AAV, and consequently, the AAV genome detected at this time point was higher (**Figure 2B**). In line with the result of the AAV genome, analysis of liver-extracted RNA reflected that AAV-IFNα and the AAV combination did not differ in the IFNα mRNA transcripts at this time point (**Figure 2C**). Moreover, three interferon-stimulated genes, interferon-stimulated gene 15 (ISG15), ubiquitin-specific peptidase 18 (USP18), and 2’-5’-oligoadenylate synthetase (2,5-OAS) were similarly induced in both experimental groups. Thus, 15 days after AAV inoculation, the expression IFNα and ISGs was similar in both experimental groups.

### Adeno-Associated Virus-L37pA Attenuates the Induction of Gene Expression Programs of Pathways Involved in Inflammation

**Figure 3:**
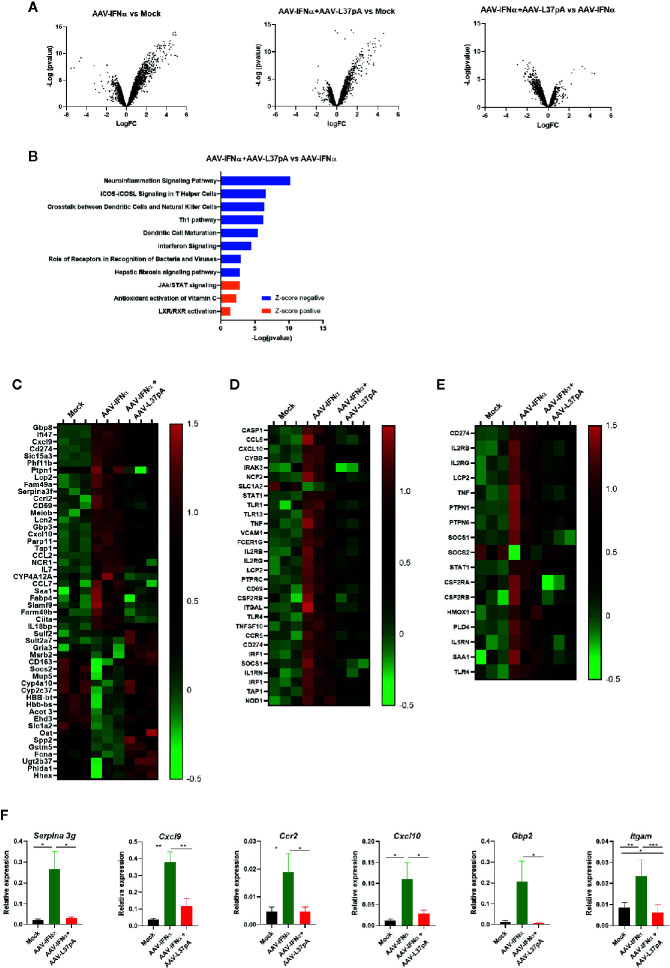
Coadministration of AAV-IFNα and AAV-L37pA modify the gene expression program modulated by AAV-IFNα in the liver. mRNA was isolated from the liver as described in **Figure 2A** and differentially expressed genes were analyzed by microarray analysis. **(A)** Volcano plot of differentially expressed genes in the liver of mice untreated (Mock), treated with AAV-IFNα or AAV-IFNα and AAV-L37pA. **(B)** Top significantly affected pathways based on Ingenuity pathway analysis (IPA). The horizontal bars denote the different pathways based on the z-score. Orange color indicates upregulation, while blue color indicates downregulation. **(C)** Heat map representing the relative expression of most differentially expressed genes. **(D)** Heat map representing the relative expression of genes contributing to the pathways with a negative Z-score. **(E)** Heat map representing the relative expression of genes contributing to the pathways with a positive Z-score. **(F)** Validation of relevant differentially expressed genes by RT-qPCR. Mean ± SEM is represented. *p < 0.05, **p < 0.001, ***p < 0.001 One-way ANOVA followed by Tukey’s multiple comparisons test.

Once we established that the livers from both experimental groups contained similar levels of AAV vector, and similar levels of IFNα and ISGs mRNAs 15 days after AAV inoculation, we extended the transcriptomic analysis using gene expression microarrays. Hundreds of genes were differentially modulated by the expression of IFNα. The Volcano plot of AAV-IFNα vs. non-injected mice was characterized by a more prominent induction of gene expression than downregulation of expression (**Figure 3A**). The combination of AAV-IFNα and AAV-L37pA induced a similar modulation of the liver transcriptome, but significant differences were detected when the AAV combination was compared with AAV-IFNα. For this analysis, transcripts were selected as significant using a B-statistic cut-off (B > 0). Following this criteria, 18,166 genes were significantly expressed between both experimental groups. The Volcano plot reflected that the greater proportion of differentially expressed genes were downregulated in the combination treatment vs. AAV-IFNα alone (**Figure 3A**). This pattern was also observed when upregulated or downregulated pathways were analyzed (**Figures 3B, C**). Seven of the 11 most significant modulated pathways had a negative Z-score, indicating that those pathways were downregulated in mice treated with AAV-IFNα and AAV-L37pA as compared to mice treated with AAV-IFNα alone (**Figures 3B, D**). The upregulated pathways highlight the mechanism of action of L37pA involving activation of nuclear receptors such as LXR/RXR and the anti-oxidative activity described previously for apoA-I mimetic peptides (28) (**Figures 3B, E**). The downregulated pathways pointed to a reduced inflammatory process since they included the pathways related to processes such as the role of receptors in recognition of bacteria and viruses, dendritic cell maturation, crosstalk between dendritic cells and natural killer cells, and Th1 pathways, among others. Several genes that predicted the activation of nuclear receptors such as LXR/RXR and PPARγ were selected for validation by RT-PCR. As shown in **Figure 3F**, *Serpina 3g*, *Cxlc9*, *Ccr2*, *Cxcl10*, *Gbp2*, and *Itgam* increased significantly in the group treated with AAV-IFNα, but the expression of these genes in the group treated with the combination was much lower with values similar to those observed in non-injected animals.

### Adeno-Associated Virus-L37pA Reduced Inflammatory Pathways Activated by Adeno-Associated Virus-Interferon-Alpha at the Protein Level

**Figure 4:**
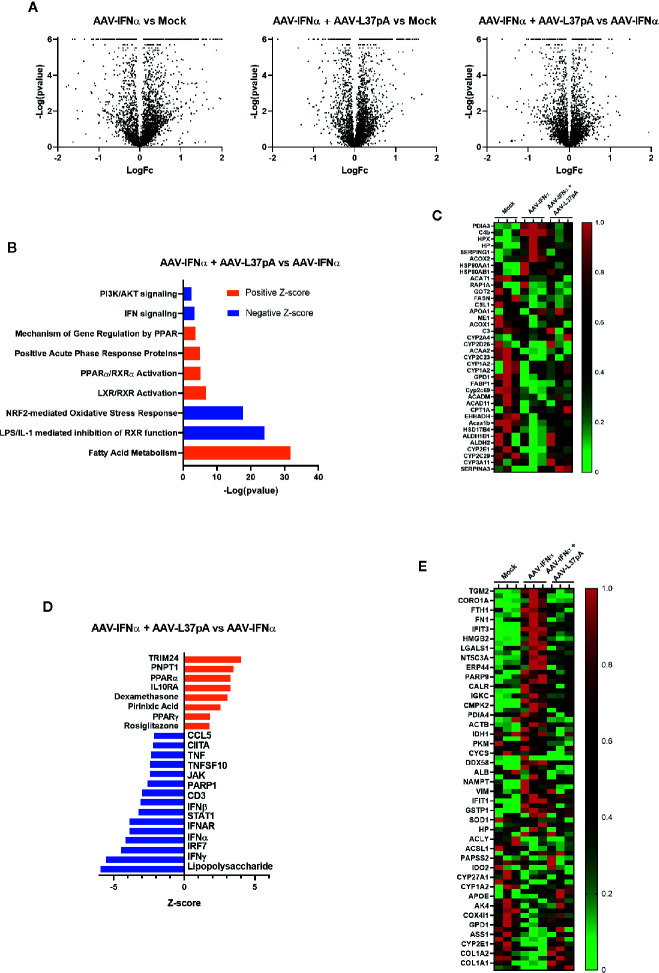
Long-term expression of L37pA modifies the AAV-IFNα effect in the liver at the protein level. Total proteins were isolated from the liver as described in **Figure 2A** and proteomic analysis was performed. **(A)** Volcano plot of proteins different levels in the liver of mice untreated (Mock), treated with AAV-IFNα or AAV-IFNα and AAV-L37pA. **(B)** Top significantly affected pathways based on Ingenuity pathway analysis (IPA). The horizontal bars denote the different pathways based on the z-score. Orange color indicates upregulation, while blue color indicates downregulation. **(C)** Heat map representing the relative protein levels contributing to the top significantly affected pathways. **(D)** Top significantly affected upstream regulators based on Ingenuity pathway analysis (IPA). The horizontal bars denote the different pathways based on the Z-score. Orange color indicates upregulation, while blue color indicates downregulation. **(E)** Heat map representing the relative protein levels contributing to the top significantly affected upstream regulators.

To validate and extend these observations at the protein level, we performed a proteomic analysis of the livers of mice treated with AAV-IFNα alone or combined with AAV-L37pA at day 15 after AAV inoculation. Overall, 3,692 proteins were identified of which 3,452 were quantified. The number of differential proteins (q < 0.05) was 1,586, 1,346, and 683 for AAV-IFNα vs control, AAV-L37pA vs control and AAV-L37pA vs AAV-IFNα contrasts, respectively (**Figure 4A**). Ingenuity pathway analysis identified significant repression of inflammatory processes such as LPS/IL-1 mediated inhibition of RXR function, NRF2-mediated oxidative stress response, IFN signaling, and PI3K/AKT signaling. Interestingly, other pathways were upregulated, including fatty acid metabolism, LXR/RXR activation, PPARα/RXRα activation, and gene regulation mechanism by PPAR (**Figure 4B**). The proteins supporting the modulation of these pathways are shown in **Figure 4C**. Except for some upregulated proteins such as PDIA3, C4b, HPX, AAV-IFNα induced a marked downregulation in the proteome (**Figure 4C**), in good agreement with the panel of upstream regulators inferred from the IPA analysis (**Figures 4D, E**). Among these regulators, downregulation of inflammatory and immune processes such as lipopolysaccharide, IFNγ, IFNα, IFNβ, IRF7, CD3, and TNF were proposed. Furthermore, stimulation of dexamethasone, IL10RA, and activation of PPARα/γ also pointed to the activation of anti-inflammatory processes.

### Coadministration of Adeno-Associated Virus-Interferon-Alpha and Adeno-Associated Virus-L37pA Do Not Alter the Liver Histology

**Figure 5:**
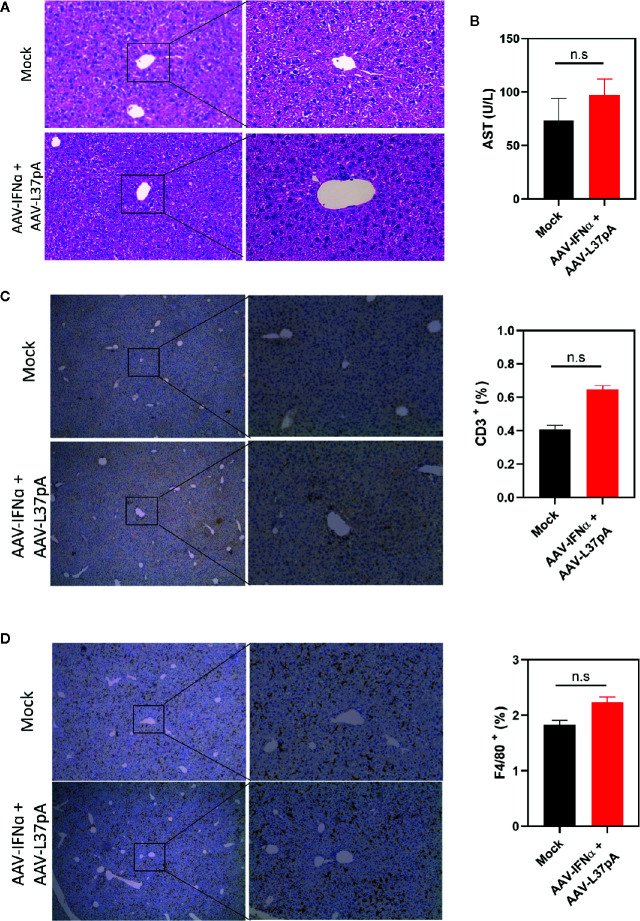
Long-term expression of AAV-IFNα and AAV-L37pA does not induce alterations in the liver. C57BL/6 mice (N = 6) were left untreated (mock) or received 1.2x10^11^ vg/mouse of AAV-IFNα combined with 9.5x10^11^ vg/mouse AAV-L37pA. Mice were followed for 120 days and then sacrificed. **(A)** The histology of the liver was analyzed by hematoxylin and Eosin staining. **(B)** Aspartate transaminase (AST) serum levels. **(C)** Representative image of intrahepatic T lymphocytes identified by immunohistochemistry against CD3 and quantification of T lymphocytes in the liver. **(D)** Representative image of intrahepatic macrophages identified by immunohistochemistry against anti-F4/80 and quantification of macrophages in the liver. Mean ± SEM is represented. n.s, non-significant. Student t-test.

**Figure 6:**
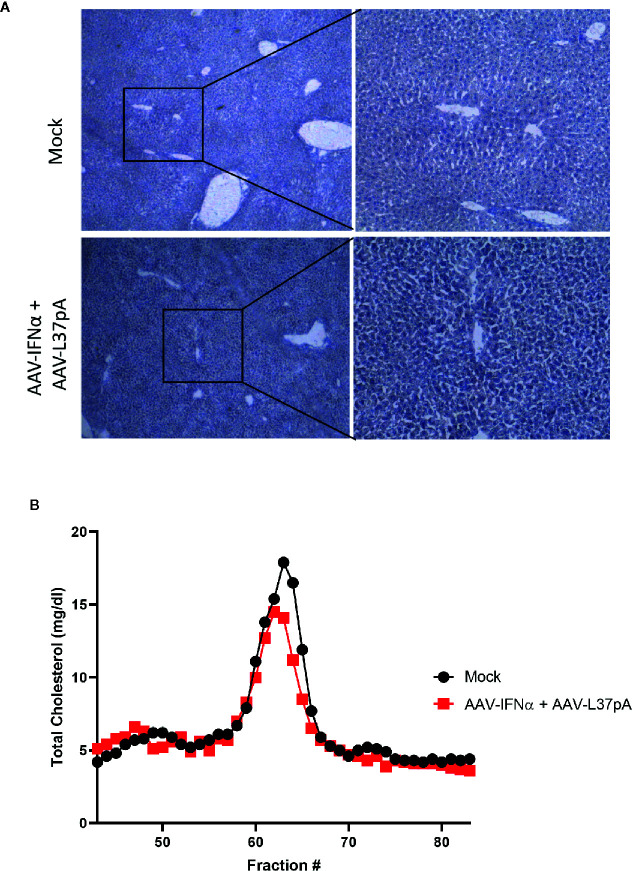
Long-term effect on lipid accumulation in the liver and on plasma lipoprotein profile. C57BL/6 mice (N = 6) were left untreated (mock) or received 1.2x10^11^ vg/mouse of AAV-IFNα combined with 9.5x10^11^ vg/mouse AAV-L37pA. Mice were followed for 120 days and then sacrificed. **(A)** Lipid accumulation in the liver was analyzed by Fat Red staining. Representative images. **(B)** Lipoprotein profile of mice after fast protein liquid chromatography followed by cholesterol determination in the different fractions.

Mice treated with the combination of AAV-IFNα and AAV-L37pA did not exhibit any signs of toxicity during the 120 days follow-up. At this time point, we analyzed the livers to check for any abnormalities in liver histology. First, hematoxylin and eosin staining of liver sections were performed. Both liver parenchyma and immune infiltration were normal and indistinguishable from those of non-injected control animals (**Figure 5A**). In line with these results, serum transaminases remained at baseline levels (**Figure 5B**). To further corroborate the absence of inflammation in the treated liver, we performed an immunohistochemical analysis to detect T lymphocytes using an anti-CD3 monoclonal antibody. Quantification of five liver slides from three mice indicated that the T cell infiltration difference was not significant between non-injected animals and the mice treated with the combination (**Figure 5C**). To evaluate macrophage infiltration into the liver, an antibody against F4/80 was used. No differences were detected, as shown in **Figure 5D**. We also evaluated the lipid accumulation in the liver using a Fat Red staining of frozen liver sections. No hints of micro- or macroestatosis were detected in the mice treated with AAV-IFNα and AAV-L37pA (**Figure 6A**). Finally, we analyzed the plasma lipoprotein profile analyzing the cholesterol content of different fractions of plasma samples from mock-treated mice or mice treated with the combination. A similar lipoprotein profile were observed in both experimental groups (**Figure 6B**).

## Discussion

The type I interferon system is the first line of defense against viruses. Detection of viral genomes by Toll-like receptor three or cytoplasmic RNA or DNA receptors induce the release of IFNβ and the different subtypes of IFNα. The secreted interferons interact with the IFNA receptor, activating an intracellular cascade that leads to the upregulation or downregulation of hundreds of genes. This transcriptional program establishes an antiviral state in the target cell. In addition. the IFNA receptor is expressed in immune cells. IFNα/β is a potent inductor of the innate immune system cells such as NK cells and is a critical cytokine bridging the innate and adaptive immune response (29). Even though all these antiviral mechanisms triggered by IFNα should be operational in mice treated with AAV-IFNα, the AAV vector encoding IFNα is able to persist as an episome in the cytoplasm of hepatocytes, releasing IFNα into the circulation for more than one month. The continuous expression of the cytokine in the circulation induces lethal hematological toxicity, as indicated by a decrease in white blood cells, red blood cells, and platelets. Interestingly, coadministration of an AAV vector encoding the apoA-I mimetic peptide L37pA prevented the lethal toxicity. The white blood cell levels were similar to those observed in mice treated with AAV-IFNα alone, probably due to the inhibition of lymphocyte egress from lymphoid organs mediated by the low but sustained levels of IFNα (30). However, the baseline levels of platelets and red blood cells were mostly preserved with the combined treatment. These results can be explained by the reduced IFNα levels observed in this experimental group compared to the mice treated with the AAV-IFNα alone. Thus, the dual AAV system is an exciting model to study the mechanisms that leads to the silencing of the AAV vector and to study the antiviral responses orchestrated by IFNα. Our genomic and proteomic analysis did not identify any potential antiviral pathway that was enhanced by the AAV combination but revealed an anti-inflammatory program that counteracts the IFNα-activated pathways. While most of the genes and proteins remained unaltered, genes and proteins of specific inflammatory pathways were downregulated. These data are consistent with reports that have found an association between chronic activation of the interferon-alpha system and a dampened antiviral function and defective T cell responses. This was first demonstrated in non-human primates infected with simian immunodeficiency virus. Non-pathogenic infection was associated with lower type I interferon signaling, while pathogenic infection progressing to AIDS was characterized by a higher type I interferon signature (31). The direct demonstration that blockade of type I interferon signaling improved the control of a viral infection was made in a mouse model of chronic lymphocytic choriomeningitis virus infection. It was shown that reduced type I interferon enhanced antiviral immunity, reduced inflammation, and decreased IL-10 and PD-L1 in myeloid cells (32–34). More recently, Lercher et al. reported that IFNα acting on hepatocytes suppresses the urea cycle, generating a microenvironment deficient in arginine and ornithine that dampens antiviral T lymphocytes (35). All in all, L37pA may enhance the antiviral activity of IFNα and the silencing of the AAV by partially abrogating the transcriptional program of this cytokine.

L37pA has been shown to interact with the scavenger class B type I receptors (SR-B1) (36). Binding of L37pA to SR-B1 leads to the peptide’s internalization and blocks the uptake of another SR-B1 ligand, such as lipopolysaccharide. Here, we show that long-term expression leads to the activation of several pathways mediated by nuclear receptors such as liver X receptor/retinoid X receptor (LXR/RXR) and peroxisome proliferator-activated receptor-gamma (PPAR) α/γ. Both nuclear receptors are essential ligand-activated receptors that regulate lipid metabolism in cells. LXR/RXR is activated by oxidized cholesterol and induces the expression of genes involved in cholesterol cell homeostasis (37). PPARα is activated by fatty acids and is essential for lipid metabolism but also exerts anti-inflammatory activities. Activation of LXR has been shown to activate the transcription of SR-B1 (38) and to reduce SR-B1 surface levels (39). PPARγ ligands also enhance both mRNA and protein levels of SR-B1 (40). However, an effect of an SR-B1 ligand on LXR or PPAR has not been previously reported. Therefore, further studies are required to establish a link between L37pA and these nuclear receptors and whether endogenous SR-B1 ligand may modulate the activity of IFNα in chronic infections. Recently, PPARγ agonists have been shown to exert an antiviral activity in a mouse model of HIV (41) and a growing body of literature has shown the interplay between the lipid metabolism and the interferon response. Lipid droplets, the major lipid storage organelles in the steady-state, are transformed in innate immune hubs that accumulate interferon-inducible proteins upon bacterial or danger signal exposure (42). One of these interferon-induced proteins is cholesterol-25-hydroxylase. This enzyme converts cholesterol to the oxysterol 25-hydroxycholesterol, promoting the cholesterol esterification for storage in lipid droplets. This process leads to a decrease in the accessible pool of cholesterol in the plasma membrane, rendering cells resistant to cholesterol-dependent cytolysins and cell-to-cell bacteria spread (43, 44). Oxysterols also induce the anti-inflammatory responses mediated by PPARγ (45) and LXR. In this case, a negative bidirectional negative crosstalk between the IFNγ signaling and LXR has been characterized (46). Our results support the relevance of the crosstalk between lipid metabolism and the IFNα. Intriguingly, the apoA-I mimetic peptides do not induce a total shutdown of the IFN-mediated transcriptional program. The expression of some interferon-stimulated genes is maintained and may be critical for the fine-tune modulation of the IFN response. This is the case of the ubiquitin-like protein ISG15. In humans, these proteins have been described as a key negative regulator of the type I IFN-mediated autoinflammation by promoting the accumulation of the negative regulator USP18 (47). In the context of AAV gene therapy, the activation of this SR-B1-mediated signaling by endogenous ligands may be a novel mechanism that mediates AAV silencing in some patients, which could be pharmacologically interfered with. Alternatively, the control of excessive inflammation to promote effective antiviral responses with apoA-I mimetic peptides may be a new application for those peptides currently in clinical trials.

## Data Availability Statement

The datasets presented in this study can be found in online repositories. The data presented in the study are deposited in the GEO repository, accession number GSE162764 and in the Proteomexchange repository, accession number PXD023281.

## Ethics Statement

The animal study was reviewed and approved by the Ethics Committee for Animal Testing of the University of Navarra.

## Author Contributions

MF-S and PB designed the experiments. MF-S, CAD-T, AB, MV, NA, and CG performed the experiments and processed the samples. SC and FC performed the proteomic analysis. PB performed all the statistical analyses. MF-S, FA, and PB analyzed the data. MF-S and PB wrote the manuscript. All authors contributed to the article and approved the submitted version.

## Funding

This study was supported by the Instituto de Salud Carlos III (PI16/00668, PI19/01128, PI20/00002) cofinanced by Fondos Feder Gobierno de Navarra Proyecto LINTERNA Ref.: 0011-1411-2020-000075 and Joint Translational Call for Proposals 2015 (JTC 2015) TRANSCAN-2 (code: TRS-2016-00000371). This project has received funding from the European Union’s Horizon 2020 research and innovation programme under the Marie Skłodowska-Curie grant agreement No 765394. FA receives a Miguel Servet I (CP19/00114) contract from ISCIII (Instituto de Salud Carlos III) co-financed by FSE (Fondo Social Europeo). AB is recipients of PFIS fellowship from ISCIII (FI20/00058), and MF-S is recipients for a fellowship of the Aid Program Assigned to Projects from the University of Navarra. The Proteomics Unit belongs to ProteoRed, PRB3-ISCIII, supported by grant PT17/0019/0001 (FC). Comunidad de Madrid Grant B2017/BMD-3817 (FC).

## Conflict of Interest

The authors declare that the research was conducted in the absence of any commercial or financial relationships that could be construed as a potential conflict of interest.
